# Mapping Hydrogen Migration Thresholds for Site-Specific HDX-MS

**DOI:** 10.1016/j.mcpro.2025.101075

**Published:** 2025-10-09

**Authors:** Charles C. Mundorff, Sarah Hadley, Lisa M. Tuttle, Yuqi Shi, Graeme C. McAlister, Rosa Viner, Rachel E. Klevit, Miklos Guttman

**Affiliations:** 1 Department of Medicinal Chemistry; University of Washington, Seattle, Washington, USA; 2 Department of Biochemistry, University of Washington, Seattle, Washington, USA; 3 Thermo Fisher Scientific, San Jose, California, USA

**Keywords:** hydrogen–deuterium exchange, HDX-MS, scrambling, hydrogen migration, site-specific

## Abstract

A long-standing limitation of Hydrogen-Deuterium Exchange Mass Spectrometry (HDX-MS) has been the difficulty in accurately measuring amide exchange with single amide resolution. Excitation of peptides or proteins during ionization, ion transmission, or collisional activation rapidly induces intermolecular hydrogen migration, leading to a loss of the deuterium-labeled state; a term commonly known as “scrambling.” Electron-based fragmentation methods in conjunction with gentle ion transmission settings can minimize scrambling but often not completely. Levels of scrambling have been shown to vary with ion transmission settings, peptide charge, and size, but the general properties that govern the susceptibility of peptides to scrambling are not well understood. Furthermore, it remains unclear whether scrambling is generally a global process or if local scrambling networks commonly exist within peptides. Here, we examine a panel of peptides using gentle electron transfer dissociation and map the activation thresholds of scrambling to define a relationship between peptide charge density and scrambling propensity. This study suggests that by and large, the scrambling process has a single activation threshold and involves all exchangeable sites within a peptide. For some peptides, the activation energy required for scrambling is surprisingly close to that of amide bond dissociation.

## Highlights

• Supplemental HCD after ETD does not impact scrambling.
• All sites within a peptide have one global threshold for scrambling.
• Thresholds for scrambling vary across peptides by size, charge, and sequence.
• Some peptides have thresholds for scrambling close to the threshold for fragmentation.

## Graphical Abstract

**Figure:**
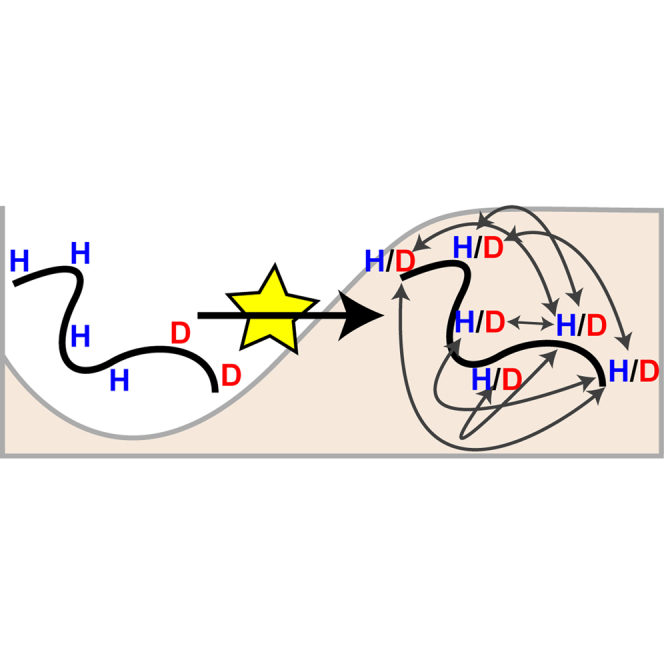

## In Brief

It has long been appreciated that collisional activation of peptides leads to rearrangement of hydrogens at exchangeable sites (termed ‘scrambling’), but the factors that govern a peptide’s susceptibility to scrambling remain undefined. Here we map the activation thresholds for scrambling across a panel of peptides to find how much the thresholds vary between peptides, and that for a given peptide there appears to be a single threshold at which point all exchangeable hydrogens start to rearrange.

HDX-MS is a widespread tool for the structural analysis of proteins and protein-ligand complexes. The most common approach for HDX-MS is bottom-up analysis, where a protein labeled with deuterium is rapidly denatured and digested under acidic conditions to generate many peptides, which are analyzed for deuterium incorporation. By measuring amide exchange kinetics across many peptides, it is possible to obtain dynamic information for the protein analyte and map interaction sites and allosteric effects with ligands. An ongoing limitation with HDX-MS is that exchange is measured on the peptide level, and achieving comprehensive single amide-level exchange is exceedingly challenging with existing methods. Early studies have attempted to derive residue-specific exchange information from the fragmentation spectra of deuterated peptides, only to find that upon collisional activation, hydrogens ‘scramble’ from the amide positions where they were labeled, to end up across all other amides, and likely exchangeable sites on sidechains and termini as well (1, 2, 3). Despite some early conflicting reports (4, 5), it has since been well-established that collisional activation cannot provide site-specific HDX measurements, with the possible exception of stapled peptides, where the conformational freedom is too restricted to allow for physical exchange of the amide deuterium to all other sites (6).

Interest in site-specific HDX-MS was revitalized by studies demonstrating that, with low ion excitation, both electron capture dissociation (ECD) and later electron transfer dissociation (ETD) can generate *c/z* ions with minimal scrambling (7, 8, 9, 10). In parallel, top-down approaches have also shown that electron-based methods are capable of producing fragmentation devoid of scrambling (11, 12). Later studies have utilized electron-based methods to investigate levels of scrambling (13, 14, 15) as well as derive mechanistic insights into HDX mechanisms (16, 17). Reporter peptides such as peptide P1 (HHHHHHIIKIIK) were developed as specific probes to directly measure the levels of scrambling (18). Due to rapid deuterium loss (back-exchange) of the histidine amides, a deuterated sample of P1, quickly diluted from D_2_O to H_2_O, can introduce the peptide in a state where only the six C-terminal amides are deuterated. This P1 peptide is commonly used to probe scrambling levels and optimize conditions on various MS platforms (19, 20, 21, 22). As an alternative, the ammonia loss fragment has also served as a reporter of scrambling as the N-terminal protons exchange rapidly and therefore will have completely back-exchanged prior to ETD/ECD analysis (23). More recent studies using newer MS platforms with gentle ion transmission settings have also demonstrated relatively low levels of scrambling for *a/x* ions with ultraviolet photodissociation (UVPD) (20, 21), and *c/z* ions generated by electron activation dissociation (EAD) (22). However, it is important to note that in all prior studies, scrambling was very rarely zero and varied widely between different charge states of the same peptide (9, 13, 15, 19, 22). Rigorous studies employing bottom-up ETD to study site-specific HDX-MS on a well-studied protein have shown partial success. A targeted study of cytochrome C was able to obtain site-specific amide exchange kinetics for only one third of the backbone amides as partial scrambling and incomplete fragment ion series confounded much of the analysis (14).

While many studies have focused on tuning MS parameters and acquisition settings to achieve the lowest levels of scrambling possible, there has been little information regarding the activation thresholds of scrambling. Early work estimated that scrambling was induced with collisional activation energy levels approximately one-third of what was required for peptide backbone fragmentation (24). Understanding how peptide size, sequence, and charge state(s) affect the susceptibility to scrambling will be important to ultimately develop a robust platform to rigorously and accurately extract HDX kinetics from peptide MS/MS data. It is generally assumed that all exchangeable sites within a peptide participate in scrambling, but this may not always be the case. Hamuro found that scrambling levels varied on different ETD fragments of neurotensin, indicating that it may have local scrambling networks (14). To address this ongoing key question, we perform gentle ETD analysis of a panel of peptides and charge states using source collisional activation to probe scrambling thresholds. The results provide a working map of the relationship between peptide charge density (size/charge), scrambling energy thresholds, and fragmentation thresholds. In all cases, scrambling was most consistent with a global event involving all exchangeable sites (backbone amides, termini, and sidechains).

## Experimental Procedures

Peptides P1 (HHHHHHIIKIIK), AHHDIVIK, and neurotensin 8 to 13 (RRPYIL) were obtained from Anaspec and bradykinin (RPPGFSPFR), angiotensin II (DRVYIHPF), and substance P (RPKPQQFFGLM-Am) were from Sigma-Aldrich. Deuterium oxide (99.99%) was from Cambridge Isotope Labs. Optima grade water and acetonitrile were from Fisher Scientific.

Peptides were resuspended in Optima LC-MS grade water and incubated in either Optima H_2_O or 95% D_2_O for 1 h at room temperature prior to experiments. Reactions were infused at 1 μl/min using a 100 μl Hamilton syringe with a syringe pump into a tee PEEK junction and diluted 1:100 with quench buffer (0.1% formic acid in Optima LC-MS grade water) delivered by a Waters Acquity BMS pump. The combined stream was delivered directly to the ESI source of a ThermoScientific Orbitrap Ascend for MS acquisition. Full scans and targeted ETD and EThcD scans were collected when the observed deuterium level of the peptide stabilized. Source activation voltage (termed ‘source fragmentation’) was adjusted from zero up to a point where the entirety of the peptide signal was lost due to complete peptide fragmentation. Other key acquisition settings are detailed in Supplemental Table S1. For MS/MS with a weak signal for most *c/z* ions, full profile data was toggled on for the Orbitrap acquisition. Peptide fragments were identified by exact mass with the help of Protein Prospector. Spectra were exported as.csv from.raw files in ThermoScientific Freestyle and deuterium incorporation were measured using a binomial fitting approach with HX-Express v3 (25) and pyHXExpress (26).

Theoretical levels for deuterium scrambling were calculated as reported previously (14). For zero scrambling, fast exchanging sites (side chains and N/C termini) were assumed to contain only the final residual level of deuterium after dilution (0.9%). For the amides, the deuterium retention was calculated based on rates from model peptides (27) and scaled to the total deuteration observed for the intact peptide. For 100% scrambling, the total deuterium content of the peptide was assumed to be distributed evenly across all exchangeable sites. Percent scrambling for *c/z* ions was calculated based on the deuterium uptake relative to the 0% and 100% theoretical values.

## Results and Discussion

As a first step to investigating the key parameters that govern scrambling, we examined the ETD spectra for the various charge states of peptide P1, by far the most well-studied model peptide to measure intramolecular hydrogen scrambling (18). Using a rapid- 100-fold dilution of a fully deuterated sample just prior to the ESI source, it is possible to fully back-exchange all deuterium at sidechains, the termini, and the histidine backbone amides, thus achieving a stable signal for peptide P1 with deuterium incorporation predominantly at the c-terminal (slow-exchanging) amide sites. The minor contributions from the residual deuterium levels (1% final) at the fast-exchanging sites only have a very minor contribution to the signal and are easily accounted for when modeling deuterium localization. The stable selectively deuterated signal enabled us to collect data for a broad range of conditions and to sample all observable charge states. Acquisition parameters on the Orbitrap Ascend for ion transmission between the source, ion funnel, ion routing multipoles, C-trap, and ion trap regions were optimized to minimize the level of ion excitation and thereby mitigate scrambling (Supplemental Table S1). Some of the optimizations resulted in lower ion transfer efficiency with an approximately 10 to 20% drop in signal intensity from standard settings. Source temperature was not a variable we examined as it was already tuned low to minimize in-source back-exchange, but it is important to note that this parameter will also likely affect scrambling (13).

**Fig. 1:**
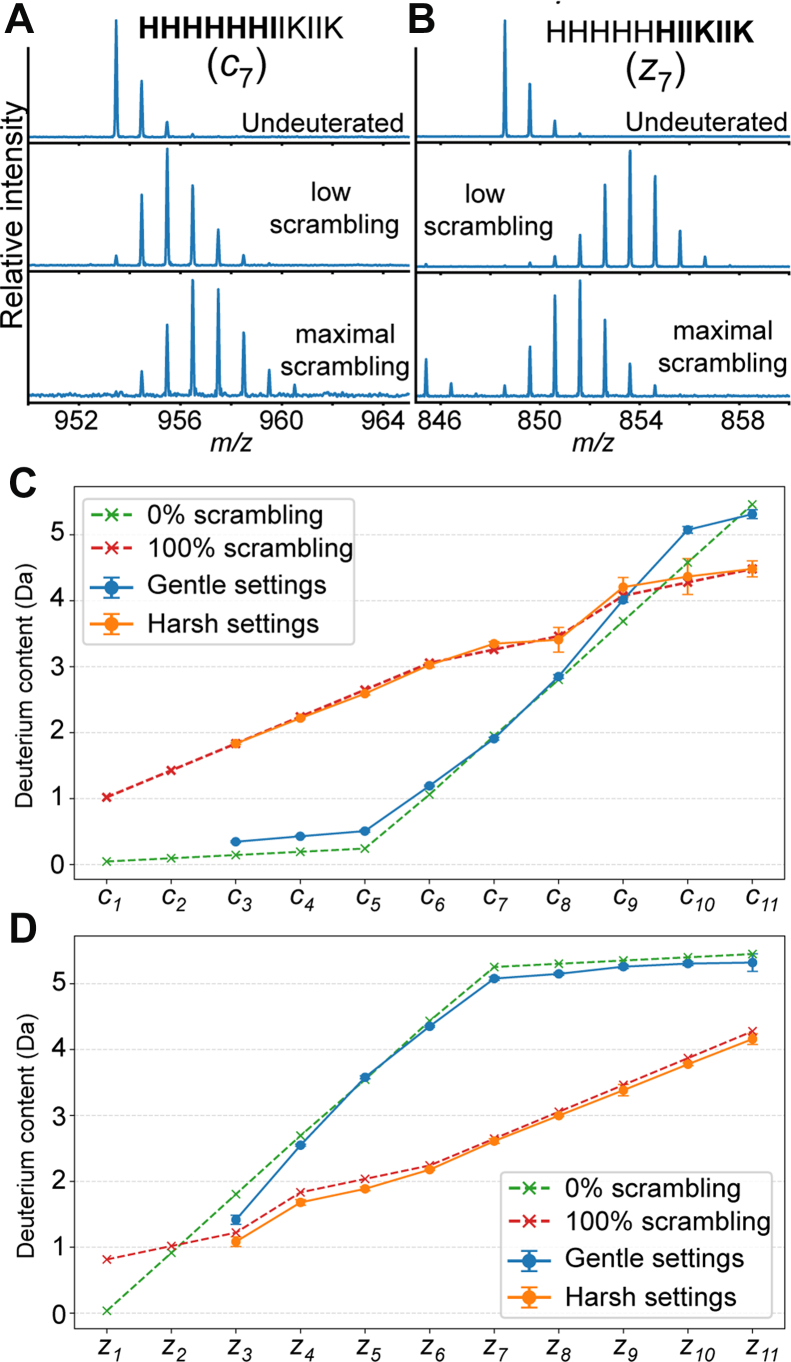
**Scrambling levels observed for the 3+ charge state of peptide P1 with optimized ion transmission settings for low scrambling (Gentle) or elevated source fragmentation energy (Harsh) to achieve maximal scrambling**. *A*, *B*, the observed deuterium uptake for the *c*_7_ and *z*_7_ ions are shown. *C*, *D*, Theoretical uptake for no scrambling (*green*) and 100% scrambling (*red*) are shown for the *c* and *z* ion series as dashed lines. The observed deuterium levels with low scrambling and 100% scrambling conditions are shown in *blue* and *orange*, respectively. *Error bars* represent standard deviations from triplicate measurements.

The 3+ precursor produced a strong signal for many *c/z* ions, which at optimal settings produced deuteration levels consistent with very low scrambling across the peptide (Fig. 1). With the explicit assumption that all amides and fast-exchanging sites are participating in the scrambling process, the level of scrambling for the *c*_*6*_ ion is 6.6% (estimated from 1.19 Da observed; 1.06 Da expected for 0% scrambling and 3.06 Da expected for 100% scrambling). Analysis of the *c*_*3*_ to *c*_*8*_ and *z*_*4*_ to *z*_*11*_ ions revealed the average level of scrambling to be 7.9%. A schematic of peptide P1 with all exchangeable sites and *c/z* ion cleavage positions is shown in Supplemental Figure S1. We note that minor assumptions, such as the location of additional protons imparting the charge, have a minor effect on the theoretical scrambling curves, and for this study, we assumed the charge to be at basic sites on the peptides with the maximum distance between charges. For example, the P1 peptide +3 charge state is assumed to have protonation at the N-terminus, central histidine sidechain, and the C-terminal lysine sidechain. The differences in the number of exchangeable sites per residue result in a non-linearity of the 100% scrambled model. For the sake of HDX-MS analysis, it is important to note that *c/z* ion cleavage occurs such that each c ion contains the backbone amide of the C-terminally adjacent residue, and *z* ions lack the N-terminal amine. The 4+ charge state of P1 had more spectral overlap for several of the *c/z* ions, which confounded deuterium incorporation analysis, but from the available set of clean fragment ion signals, the average scrambling level was 19.9%.

Through the course of comparing charge states, it was noted that several ETD fragment ions were observed as combinations of *c* and *c*-1 or *z* and *z* + 1 ions. Hydrogen transfer products are not uncommon for electron-based dissociation (28, 29), but are particularly important to account for when performing HDX-MS as the hydrogen transfer shifts the reference isotopic profile. For peptide P1, the different charge states displayed varying levels of c vs. c-1 and z vs. z + 1 profiles (Supplemental Fig. S2*A*). Therefore, an undeuterated sample was collected using identical acquisition parameters to serve as the reference for the corresponding deuterium incorporation analysis to ensure no confounding effects could be attributed to differences in the levels of hydrogen transfer products. It is also important to consider the mechanism of the hydrogen transfer products as the process may shift deuterium from amide positions as well. However, prior mechanistic studies using isotopically labeled peptides have observed that hydrogen transfer products largely result from the alpha and sidechain hydrogens, and not the amide hydrogen (30, 31).

### ETD With Supplemental Activation

**Fig. 2:**
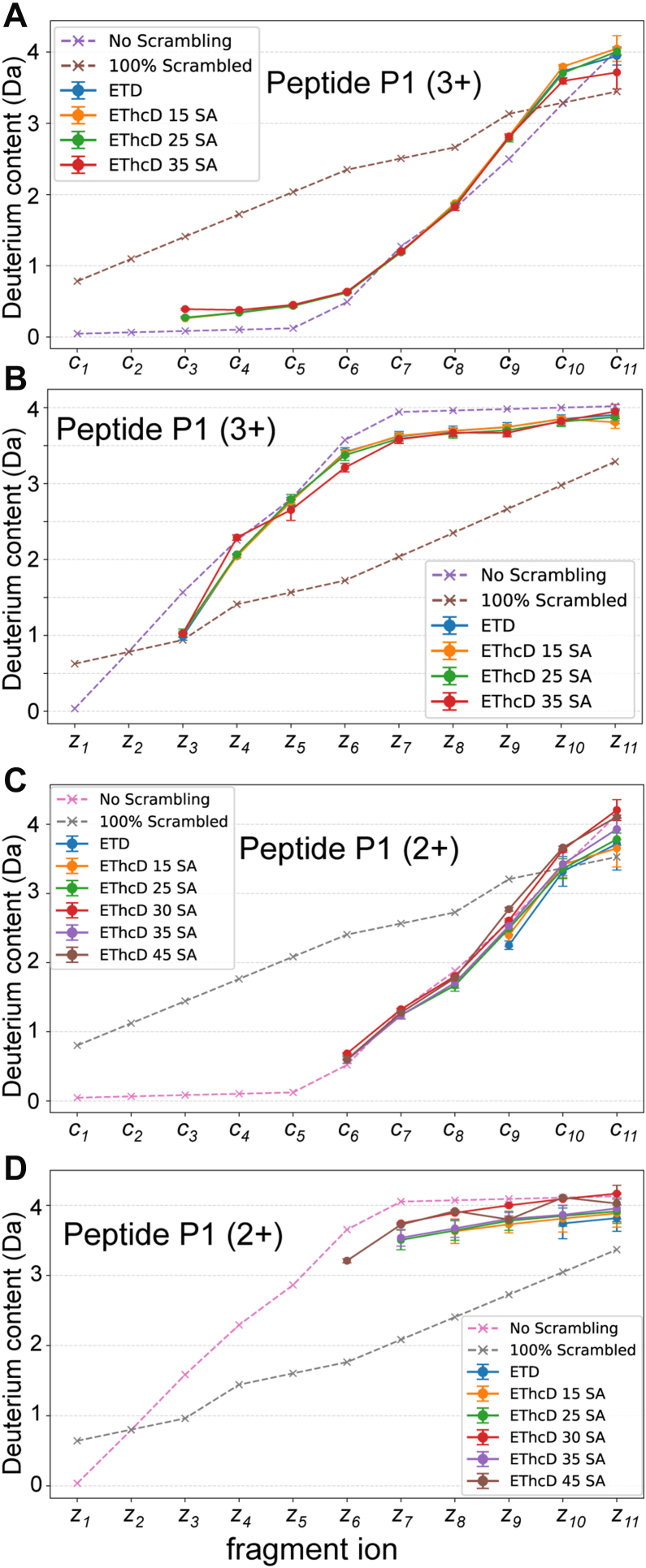
**Scrambling levels for the c and z ion series for peptide P1 using either ETD or EThcD with varying levels of supplemental activation (SA) with HCD for the 3+ precursor (*A*, *B*) and the 2+ precursor ions (*C*, *D*)**. Theoretical curves for no scrambling and 100% scrambling are shown as *pink* and *gray dashed lines*.

ETD analysis of the 2+ charge state of peptide P1 yielded very few observable *c*/*z* ions, precluding scrambling analysis. Supplemental activation with higher-energy collisional dissociation (HCD; which combines to EThcD) have been shown to dissociate any fragmentation products remaining non-covalently associated (ETnoD) to produce higher yields of *c/z* ions (32). As expected, higher levels of supplemental HCD yielded far more observable *c/z* ions for the 2+ precursor (Supplemental Fig. S3). For the 3+ precursor, EThcD *c/z* fragmentation yield was not significantly improved over ETD without supplemental activation. Prior work has utilized post-ETD collisional activation in a T-wave ion guide to enhance *c/z* ion yield without impacting scrambling levels (15). To test the potential impacts of supplemental HCD on scrambling, we performed a side-by-side comparison of the performance of ETD vs. EThcD. For both 3+ and 2+ precursors, there was no apparent increase in the level of scrambling with any level of supplemental HCD (Fig. 2). Therefore, ETnoD products can be dissociated with HCD without inducing scrambling between the non-covalently associated products. Higher levels of supplemental HCD did result in the generation of more intense *b* and *y* ions, which in a few cases led to spectral overlap with *c/z* ions to complicate deuterium incorporation analysis. Therefore, the supplemental HCD energy for EThcD was kept below 40% normalized collision energy to minimize spectral interference. Based on the *c*_*6*_ ion in the EThcD spectrum, the extent of scrambling for the 2+ precursor was 7.4%.

### Measuring Scrambling Thresholds Using Source Activation

**Fig. 3:**
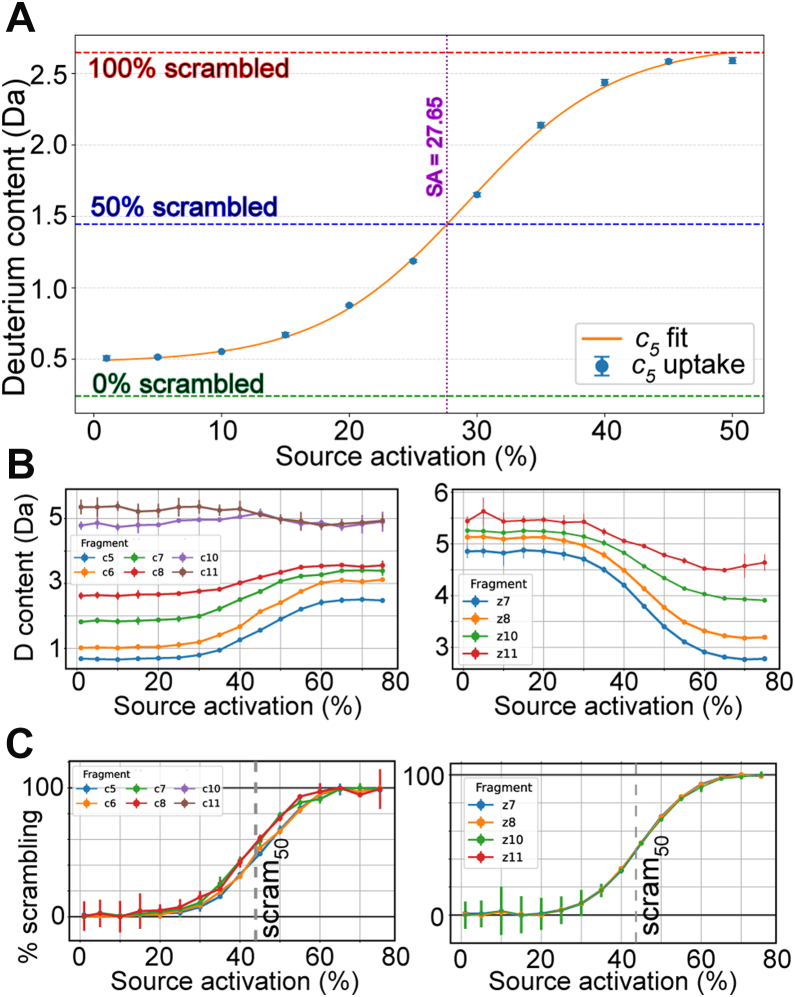
**Determining the activation thresholds for scrambling through source fragmentation ramping.***A*, the deuterium uptake for the *c*_5_ fragment ion of the 3+ charge state of peptide P1 with ETD is shown for a range of source fragmentation settings. Theoretical values for 0% and 100% scrambling are shown as green and red dashed lines. A sigmoidal fit is used to calculate the source fragmentation energy for the point at where 50% scrambling is achieved (‘scram_50_’). *B*, deuterium uptake through varied source energy is shown for all observable c/z ions. The deuterium uptake values were scaled to relative scrambling %, and the overlays are shown in (*C*). Scram_50_ curves for 2+ and 4+ charge states are shown in Figure S5.

To measure the thresholds of scrambling, we systematically measured the effect of raising the source collisional activation voltage to induce vibrational excitation prior to mass isolation and ETD. For peptide P1 we measured the level of scrambling for all observable *c/z* ions across a wide range of source activation voltages to find an apparent sigmoidal relationship reminiscent of a titration curve. An example is shown for the *c*_5_ ETD product ion from the 3+ precursor in Figure 3*A*. The curve has a stable low scrambling baseline and a maximum-scrambling plateau that was consistent with the theoretical 100% scrambled state. The sigmoidal fit was used to calculate the source activation necessary to induce 50% maximal scrambling, which we term the ‘scram_50_’. Interestingly, the scram_50_ for all observable *c* and *z* ion was remarkably consistent (Fig. 3, *B* and *C*). The fact that there was no difference between the activation threshold of scrambling among the c/z fragments strongly suggests that there is a *single global threshold* for a given precursor ion to enable hydrogen migration between *all* exchangeable sites. If certain amides were more susceptible to scrambling than others, we would have expected to see differences in the scram_50_ for a subset of *c/z* ions.

Comparison of the 2+, 3+, and 4+ charge states of peptide P1 revealed that the threshold of scrambling was very different for the different charge states, with the higher charge states having much lower scram_50_ values. This is entirely consistent with earlier reports showing higher levels of basal scrambling for higher charge states (9, 22). More importantly, within each charge state, all of the c/z ions showed a consistent scram_50_. This further corroborates the notion that there is a global energetic threshold for scrambling that involves all exchangeable hydrogens. If any preferred scrambling networks existed within peptides, then we would have expected to observe either 1) a multi-phase sigmoidal curve or 2) not all *c/z* ions showing the same threshold. Overall, these observations strongly argue that when there is sufficient vibrational excitation to initiate scrambling, all amide hydrogens start to migrate.

The series of spectra with progressive levels of source-induced scrambling was also used to assess whether, on a single molecule level, the scrambling process was gradual or abrupt. In other words, for a single ion is scrambling either zero or 100% and only due to population weighted averaging, does scrambling appear progressive/gradual. If scrambling was an all-or-nothing process, then we would expect to see some evidence of isotopic broadening. To directly test this, we closely examined the *c*_5_ ion from peptide P1, which, among all the analytes, is the fragment that should show the largest difference in deuteration between minimal and maximal scrambling. From the binomial fitting approach, a single distribution was able to describe the observed isotopic distributions sufficiently (Supplemental Fig. S4*A*). We also attempted a bimodal fit, where the fit is a best-fit combination of the minimal and maximal scrambled isotopic distributions, which was clearly inconsistent with the data (Supplemental Fig. S4*B*). This additional analysis strongly indicates that scrambling, even on a single-molecule level, is a progressive/gradual process.

### Scrambling Thresholds Across Different Peptides

To examine whether the trends observed for P1 were general across different peptides, we analyzed a panel of peptides, including common MS peptide reagents: bradykinin, substanceP, angiotensin II, neurotensin 8 to 13, and RVVPV. While these peptides were not engineered to retain deuterium solely on a specific subset of amides, we could rely on the small *c* (*c*_2_ and *c*_3_) and large z ions to assess levels of scrambling (by monitoring the transfer of D from the amides to the N-termini), as the N-terminal end of the peptides will be fully back-exchanged (1, 33, 34). Additionally, we also included a designed peptide: AHHDIVIK. Similar to P1, this was designed to only retain deuterium on the C-terminal amide positions after dilution from D_2_O and provided a smaller peptide and predominantly the 2+ and 3+ charge states.

Analysis of peptide AHHDIVIK showed trends similar to P1. The 3+ charge state had a much lower scram_50_ than the 2+ (Supplemental Fig. S5). Furthermore, all observable *c* fragments for a given charge state had the same inflection point, indicating that all sites have the same activation threshold for scrambling. Though the fragments were more limited, the same was also true when looking at the scram_50_ curves for angiotensin II (2+), bradykinin (2+), and both 3+ and 2+ charge states of substance P (Supplemental Fig. S5). Peptide RVVPV provided only one strong fragment (*c*_2_) ion, which showed no change in deuterium level with increasing source activation energy (Supplemental Fig. S6*A*). A closer examination of the spectra showed a very weak *c*_1_ ion that also did not shift with source activation energy (Supplemental Fig. S6*B*). Notably, RVVPV (2+) was the lowest *m/z* peptide analyzed in this panel and may reflect a limit as to what size peptide can be analyzed without complete scrambling.

The scram_50_ curves for neurotensin 8 to 13 (RRPYIL; 2+) also showed consistent inflection points for the *c*_3_ and *c*_4_ ions (Supplemental Fig. S6*C*). The *c*_5_ ion was also observed but showed no change, likely because the deuterium content with minimal and maximal scrambling result in similar values. We also closely examined the EThcD products corresponding to neutral losses including: the loss of the N-terminus (−17) and arginine sidechain cleavage products corresponding to −44, −59, and −101 neutral losses (35). While the signal was relatively weak, qualitatively the positions where the deuterium levels drop (above 30) is approximately in agreement with the scram_50_ observed for the *c*_3_ and *c*_4_ ions (Supplemental Fig. S6*D*). This peptide is similar to neurotensin 9 to 13 (RPYIL), one of the few peptides that has been studied closely for scrambling pathways (14). In contrast to the prior study, we observe no deuterium retention attributed to arginine sidechains, as both the overall level of deuterium was consistent with deuteration solely at the backbone amides, and the fact that all arginine sidechain neutral losses showed a drop in deuterium content with increasing scrambling, just like the N-terminus.

A far larger contrast to the earlier study is that we see no strong evidence of multiple scrambling networks or independent processes, as all available fragment ions show consistent scram_50_ profiles. Admittedly, it is possible that sub-networks of scrambling exist, but with such low energy thresholds that they are nearly completely scrambled prior to any source activation we apply. In principle, the differences between the two studies may relate to the conclusions relying on the measured vs. predicted deuterium content across different sites vs. the relationship of collisional activation to the degree of scrambling. There are also differences regarding the additional arginine on our analyte peptide, differences in the MS instrumentation, along with sampling differences, which could all explain the different outcomes. Lastly, we also note that the prior study also saw a sigmoidal effect on scrambling levels by modulating the mass isolation width for precursor selection in the ion trap to induce sideband excitation, which resembles our scram_50_ curves. We instead used quadrupole isolation, for which much narrower *m/z* widths can be selected without inducing scrambling (13).

**Fig. 4:**
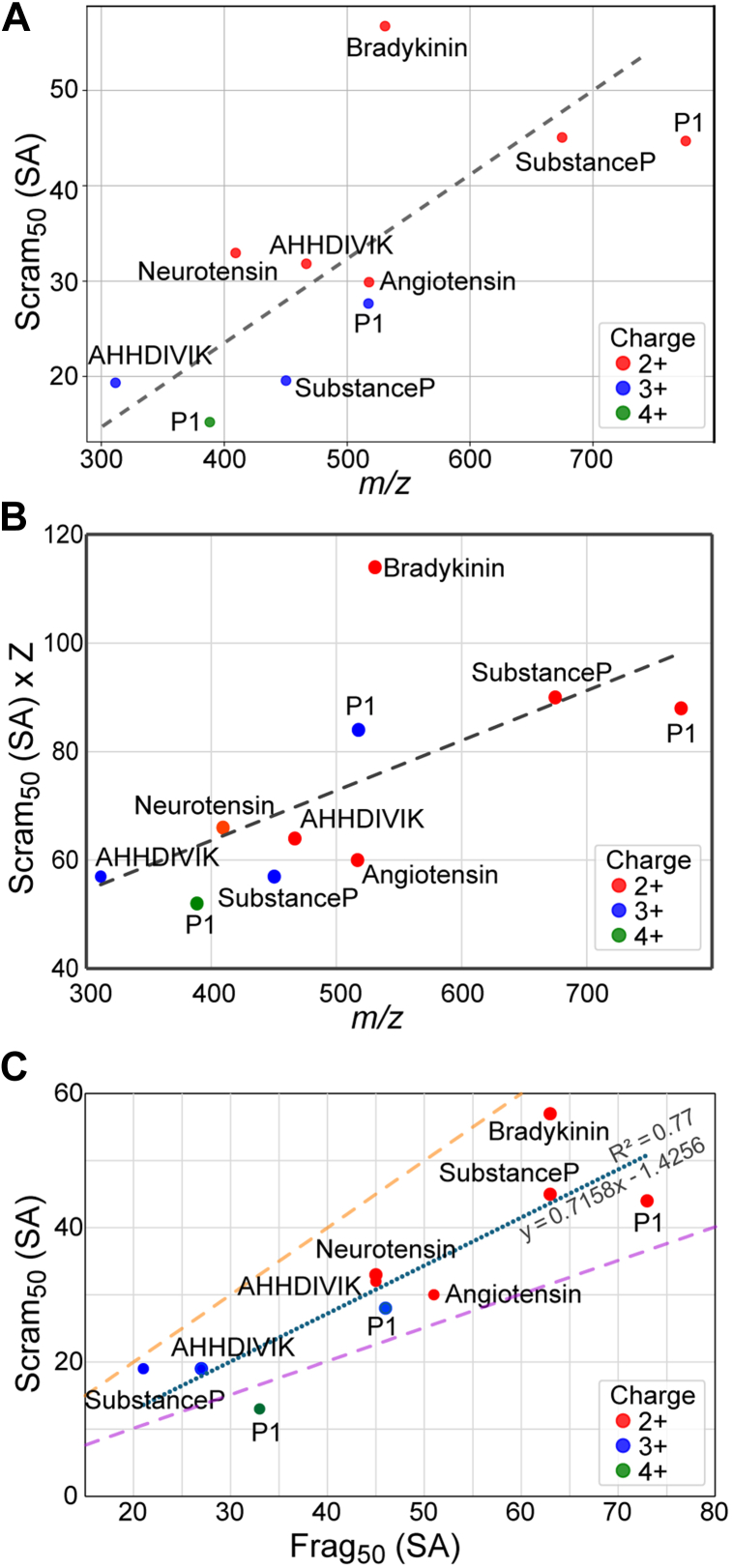
**Summary of scrambling thresholds.***A*, Relationship between the source activation energy for 50% scrambling (scram_50_) for the panel of peptides vs. their *m/z*. Colors show charge states, and peptides are labeled in the inset. *B*, the lab-frame energy (scram_50_ x charge state) is shown for the same set of peptides, indicating that different charge states for several peptides are inherently different with regard to their susceptibility to scrambling. *C*, relationship between scram_50_ and the source fragmentation energy for 50% fragmentation (frag_50_). The *dotted line* represents the linear trends with statistics shown in the inset. *Dashed lines* show positions of frag_50_ = scram_50_ (*orange*) and frag_50_=2·scram_50_ (*purple*) for visual reference.

Through the comprehensive analysis of the panel of peptides we were able to map a general relationship between the peptide charge density (*m/z*) and the scram_50_ (Fig. 4*A*). While there is generally a trend with larger *m/z* having higher scram_50_, there already exist some notable outliers. For example, bradykinin has a relatively high scram_50_ (57), far larger than angiotensin II (30), even though it has nearly the same charge density. Both peptides have arginines, and therefore have highly basic sites to hold charge. It is possible that bradykinin, having three prolines, as opposed to angiotensin II, which only has one, makes bradykinin more structurally rigid to explain the higher scram_50_. We also examined the lab-frame activation energetics to account for the inherent differences in excitation with different charge states (Fig. 4*B*). With this correction, some charge states now look similar in their scram_50_, but others are still drastically different. For example, the 2+ and 3+ of peptide P1 are now close, but the 4+ still has a far lower threshold for scrambling. Similarly, the 2+ and 3+ charge states of substanceP show drastic differences in the corrected scram_50_. Overall, while there exists a loose relationship with *m/z*, we expect that other factors, such as the gas-phase structure(s) of peptides, also highly influence scram_50_.

Lastly, we examined the relationship between the scram_50_ and the threshold for fragmentation. By monitoring the intensity of the MS/MS spectra at different source activation voltages, it is possible to estimate how much energy is required to achieve 50% fragmentation (frag_50_). A fairly strong correlation between scram_50_ and frag_50_ was observed (R^2^ = 0.77) (Fig. 4*C*). The general trend that emerges is that the scram_50_ is nearly always between 50% and 100% of frag_50,_ meaning there is not a tremendous difference in the excitation requirements between scrambling and amide bond fragmentation. In fact, there are two examples, bradykinin (2+) and substanceP (3+), for which the scram_50_ and frag_50_ are close. These two particular outliers may explain some observations in earlier literature that suggested incomplete scrambling with collisional-based fragmentation (2, 3, 36, 37, 38, 39). Both of these peptides contain prolines, which are known to be the most scissile amide bond (40, 41), which at least partially explains why their frag_50_ are relatively low. However, angiotensin II, which also contains a proline, has a larger difference between scram_50_ and frag_50_, so the presence of proline residues is not a sufficient explanation for this pattern. The outliers may also present a potential caveat for DIA approaches for bottom-up HDX-MS utilizing collisional activation, as a key assumption for the analysis is that complete and uniform scrambling will occur prior to amide bond dissociation (42, 43).

## Data Availability

Raw data has been deposited to the ProteomeXchange PRIDE server under accession number PXD066803.

## Supplemental Data

This article contains supplemental data.

## Conflict of Interests

The authors declare the following financial interests/personal relationships which may be considered as potential competing interests:

Y. S., G. M. and R. V. are employees of *Thermo Fisher Scientific*.

## References

[1] Hamuro Y., Tomasso J.C., Coales S.J. (2008). A simple test to detect Hydrogen/Deuterium scrambling during gas-phase peptide fragmentation. Anal. Chem..

[2] Jorgensen T.J., Gardsvoll H., Ploug M., Roepstorff P. (2005). Intramolecular migration of amide hydrogens in protonated peptides upon collisional activation. J. Am. Chem. Soc..

[3] Ferguson P.L., Pan J., Wilson D.J., Dempsey B., Lajoie G., Shilton B. (2007). Hydrogen/Deuterium scrambling during quadrupole time-of-flight MS/MS analysis of a zinc-binding protein domain. Anal. Chem..

[4] Johnson R.S., Krylov D., Walsh K.A. (1995). Proton mobility within electrosprayed peptide ions. J. Mass Spectrom..

[5] Deng Y., Pan H., Smith D.L. (1999). Selective isotope labeling demonstrates that hydrogen exchange at individual peptide amide linkages can be determined by collision-induced dissociation mass spectrometry. J. Am. Chem. Soc..

[6] Code C., Qiu D., Solov'yov I.A., Lee J.G., Shin H.C., Roland C. (2023). Conformationally restricted glycopeptide backbone inhibits gas-phase H/D scrambling between glycan and peptide moieties. J. Am. Chem. Soc..

[7] Zubarev R.A., Kelleher N.L., McLafferty F.W. (1998). Electron capture dissociation of multiply charged protein cations. A nonergodic process. J. Am. Chem. Soc..

[8] Zehl M., Rand K.D., Jensen O.N., Jørgensen T.J.D. (2008). Electron transfer dissociation facilitates the measurement of deuterium incorporation into selectively labeled peptides with single residue resolution. J. Am. Chem. Soc..

[9] Rand K.D., Adams C.M., Zubarev R.A., Jorgensen T.J. (2008). Electron capture dissociation proceeds with a low degree of intramolecular migration of peptide amide hydrogens. J. Am. Chem. Soc..

[10] Rand K.D., Zehl M., Jensen O.N., Jørgensen T.J.D. (2009). Protein hydrogen exchange measured at single-residue resolution by electron transfer dissociation mass spectrometry. Anal. Chem..

[11] Wang G., Abzalimov R.R., Bobst C.E., Kaltashov I.A. (2013). Conformer-specific characterization of nonnative protein states using hydrogen exchange and top-down mass spectrometry. Proc. Natl. Acad. Sci..

[12] Pan J., Han J., Borchers C.H., Konermann L. (2009). Hydrogen/deuterium exchange mass spectrometry with top-down electron capture dissociation for characterizing structural transitions of a 17 kDa protein. J. Am. Chem. Soc..

[13] Landgraf R.R., Chalmers M.J., Griffin P.R. (2012). Automated Hydrogen/Deuterium exchange electron transfer dissociation high resolution mass spectrometry measured at single-amide resolution. J. Am. Soc. Mass. Spectrom..

[14] Hamuro Y. (2017). Regio-selective intramolecular Hydrogen/Deuterium exchange in gas-phase electron transfer dissociation. J. Am. Soc. Mass. Spectrom..

[15] Rand K.D., Pringle S.D., Morris M., Engen J.R., Brown J.M. (2011). ETD in a traveling wave ion guide at tuned Z-Spray ion source conditions allows for site-specific Hydrogen/Deuterium exchange measurements. J. Am. Soc. Mass. Spectrom..

[16] Hamuro Y., Yen S. (2018). Determination of backbone amide hydrogen exchange rates of cytochrome c using partially scrambled electron transfer dissociation data. J. Am. Soc. Mass. Spectrom..

[17] Huang R.Y.C., Hudgens J.W. (2013). Effects of desialylation on human α1-Acid glycoprotein–ligand interactions. Biochemistry.

[18] Rand K.D., Jørgensen T.J. (2007). Development of a peptide probe for the occurrence of hydrogen (1H/2H) scrambling upon gas-phase fragmentation. Anal. Chem..

[19] Wollenberg D.T.W., Pengelley S., Mouritsen J.C., Suckau D., Jorgensen C.I., Jorgensen T.J.D. (2020). Avoiding H/D scrambling with minimal ion transmission loss for HDX-MS/MS-ETD analysis on a high-resolution Q-TOF mass spectrometer. Anal. Chem..

[20] Mistarz U.H., Bellina B., Jensen P.F., Brown J.M., Barran P.E., Rand K.D. (2018). UV photodissociation mass spectrometry accurately localize sites of backbone deuteration in peptides. Anal. Chem..

[21] Modzel M., Wollenberg D.T.W., Trelle M.B., Larsen M.R., Jørgensen T.J.D. (2021). Ultraviolet photodissociation of protonated peptides and proteins can proceed with H/D scrambling. Anal. Chem..

[22] Anacleto J., Kabir E., Blanco M., Leblanc Y., Lento C., Wilson D.J. (2025). Efficient, zero scrambling fragmentation of deuterium labeled peptides on the ZenoToF 7600 electron activated dissociation platform. J. Am. Soc. Mass. Spectrom..

[23] Rand K.D., Zehl M., Jensen O.N., Jørgensen T.J. (2010). Loss of ammonia during electron-transfer dissociation of deuterated peptides as an inherent gauge of gas-phase hydrogen scrambling. Anal. Chem..

[24] Duchateau M., Jørgensen T.J.D., Robine O., Nicol E., Malosse C., Chamot-Rooke J. (2014). Ion source parameters and hydrogen scrambling in the ECD of selectively deuterated peptides. Int. J. Mass Spectrom..

[25] Tuttle L.M., James E.I., Georgescauld F., Wales T.E., Weis D.D., Engen J.R. (2025). Rigorous analysis of multimodal HDX-MS spectra. J. Am. Soc. Mass. Spectrom..

[26] Tuttle L.M., Klevit R.E., Guttman M. (2025). A framework for automated multimodal HDX-MS analysis. bioRxiv.

[27] Nguyen D., Mayne L., Phillips M.C., Walter Englander S. (2018). Reference parameters for protein hydrogen exchange rates. J. Am. Soc. Mass. Spectrom..

[28] Bythell B.J. (2013). To jump or not to jump? Cα hydrogen atom transfer in post-cleavage radical-cation complexes. J. Phys. Chem. A.

[29] Peters-Clarke T.M., Riley N.M., Westphall M.S., Coon J.J. (2022). Practical effects of intramolecular hydrogen rearrangement in electron transfer dissociation-based proteomics. J. Am. Soc. Mass. Spectrom..

[30] Savitski M.M., Kjeldsen F., Nielsen M.L., Zubarev R.A. (2007). Hydrogen rearrangement to and from radical z fragments in electron capture dissociation of peptides. J. Am. Soc. Mass. Spectrom..

[31] Chung T.W., Hui R., Ledvina A., Coon J.J., Tureček F. (2012). Cascade dissociations of peptide cation-radicals. Part 1. Scope and effects of amino acid residues in Penta-, Nona-, and decapeptides. J. Am. Soc. Mass. Spectrom..

[32] Swaney D.L., McAlister G.C., Wirtala M., Schwartz J.C., Syka J.E., Coon J.J. (2007). Supplemental activation method for high-efficiency electron-transfer dissociation of doubly protonated peptide precursors. Anal. Chem..

[33] Bai Y., Milne J.S., Mayne L., Englander S.W. (1993). Primary structure effects on peptide group hydrogen exchange. Proteins.

[34] Hamuro Y. (2021). Tutorial: chemistry of Hydrogen/Deuterium exchange mass spectrometry. J. Am. Soc. Mass. Spectrom..

[35] Cooper H.J., Hudgins R.R., Håkansson K., Marshall A.G. (2002). Characterization of amino acid side chain losses in electron capture dissociation. J. Am. Soc. Mass. Spectrom..

[36] Hoerner J.K., Xiao H., Dobo A., Kaltashov I.A. (2004). Is there hydrogen scrambling in the gas phase? Energetic and structural determinants of proton mobility within protein ions. J. Am. Chem. Soc..

[37] Buijs J., Hagman C., Håkansson K., Richter J.H., Håkansson P., Oscarsson S. (2001). Inter- and intra-molecular migration of peptide amide hydrogens during electrospray ionization. J. Am. Soc. Mass. Spectrom..

[38] Monson de Souza B., Palma M.S. (2008). Monitoring the positioning of short polycationic peptides in model lipid bilayers by combining hydrogen/deuterium exchange and electrospray ionization mass spectrometry. Biochim. Biophys. Acta..

[39] Demmers J.A., Rijkers D.T., Haverkamp J., Killian J.A., Heck A.J. (2002). Factors affecting gas-phase deuterium scrambling in peptide ions and their implications for protein structure determination. J. Am. Chem. Soc..

[40] Wysocki V.H., Tsaprailis G., Smith L.L., Breci L.A. (2000). Mobile and localized protons: a framework for understanding peptide dissociation. J. Mass. Spectrom..

[41] Raulfs M.D., Breci L., Bernier M., Hamdy O.M., Janiga A., Wysocki V. (2014). Investigations of the mechanism of the "proline effect" in tandem mass spectrometry experiments: the "pipecolic acid effect". J. Am. Soc. Mass. Spectrom..

[42] Percy A.J., Slysz G.W., Schriemer D.C. (2009). Surrogate H/D detection strategy for protein conformational analysis using MS/MS data. Anal. Chem..

[43] Filandr F., Sarpe V., Raval S., Crowder D.A., Khan M.F., Douglas P. (2024). Automating data analysis for hydrogen/deuterium exchange mass spectrometry using data-independent acquisition methodology. Nat. Commun..

