# Supplementary material for: Mapping Hydrogen Migration Thresholds for Site-Specific HDX-MS
Source: Mol Cell Proteomics. 2025 Oct 9;24(11):101075. doi: 10.1016/j.mcpro.2025.101075 (PMC12637231; doi:10.1016/j.mcpro.2025.101075)
Supplement: Supplementary File [file mmc1.docx]

Supplementary Information

Mapping Hydrogen Migration Thresholds for Site-Specific HDX-MS

Charles C. Mundorff^1^, Sarah Hadley^1^, Lisa M. Tuttle^2^, Yuqi Shi^3^, Graeme McAlister^3^, Rosa Viner^3^, Rachel E. Klevit^2^, and Miklos Guttman^1^*

^1^Department of Medicinal Chemistry; University of Washington, Seattle, WA 98195

^2^Department of Biochemistry, University of Washington, Seattle, WA 98195

^3^Thermo Fisher Scientific, San Jose, CA 95134

*email: mguttman@uw.edu

Contents:

**Figure S1:** Schematic of Peptide P1 and AHHDIVIK

**Figure S2:** Hydrogen transfer products with ETD

**Figure S3:** ETD vs. EThcD fragmentation of the 2+ charge state of peptide P1

**Figure S4:** Isotopic envelopes are shown for the *c*_5_ ion from the ETD spectra

**Figure S5:** Scram_50_ analysis for the panel of peptides in this study.

**Figure S6:** EThcD analysis of peptide RVVPV and neurotensin 8-13

**Figure S7:** Mapping of fragmentation thresholds for the panel of peptides

**Table S1:** Optimized ion transmission settings for minimal scrambling on the Thermo Orbitrap Ascend


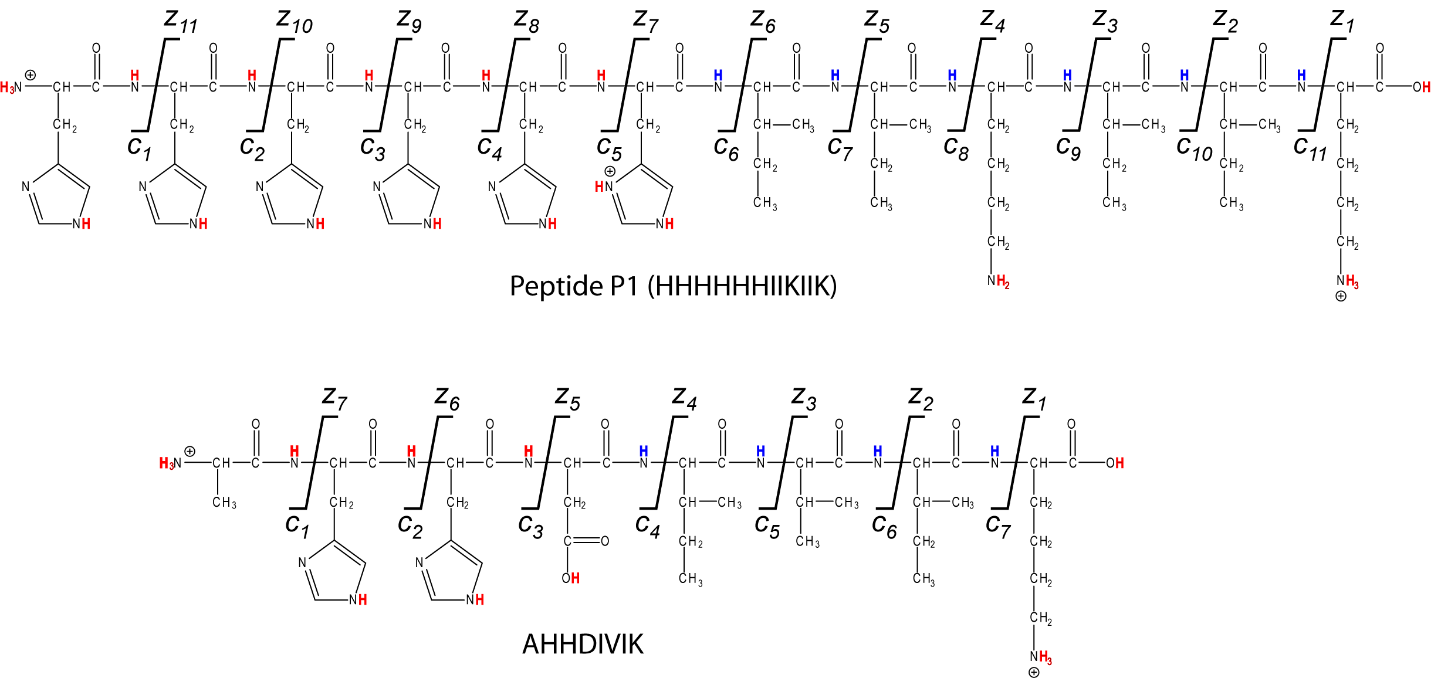


**Figure S1:** Schematic of Peptide P1 and AHHDIVIK showing the locations of the fast exchanging and slow-exchanging hydrogens (red and blue, respectively). Positions of backbone cleavage along with the resulting *c* and *z* ions are shown for each peptide. We note that the peptides were designed for the amides at the N-terminal half of the peptide to have rapid amide exchange kinetics and will contain negligible deuterium after the 100-fold dilution into quench solution.


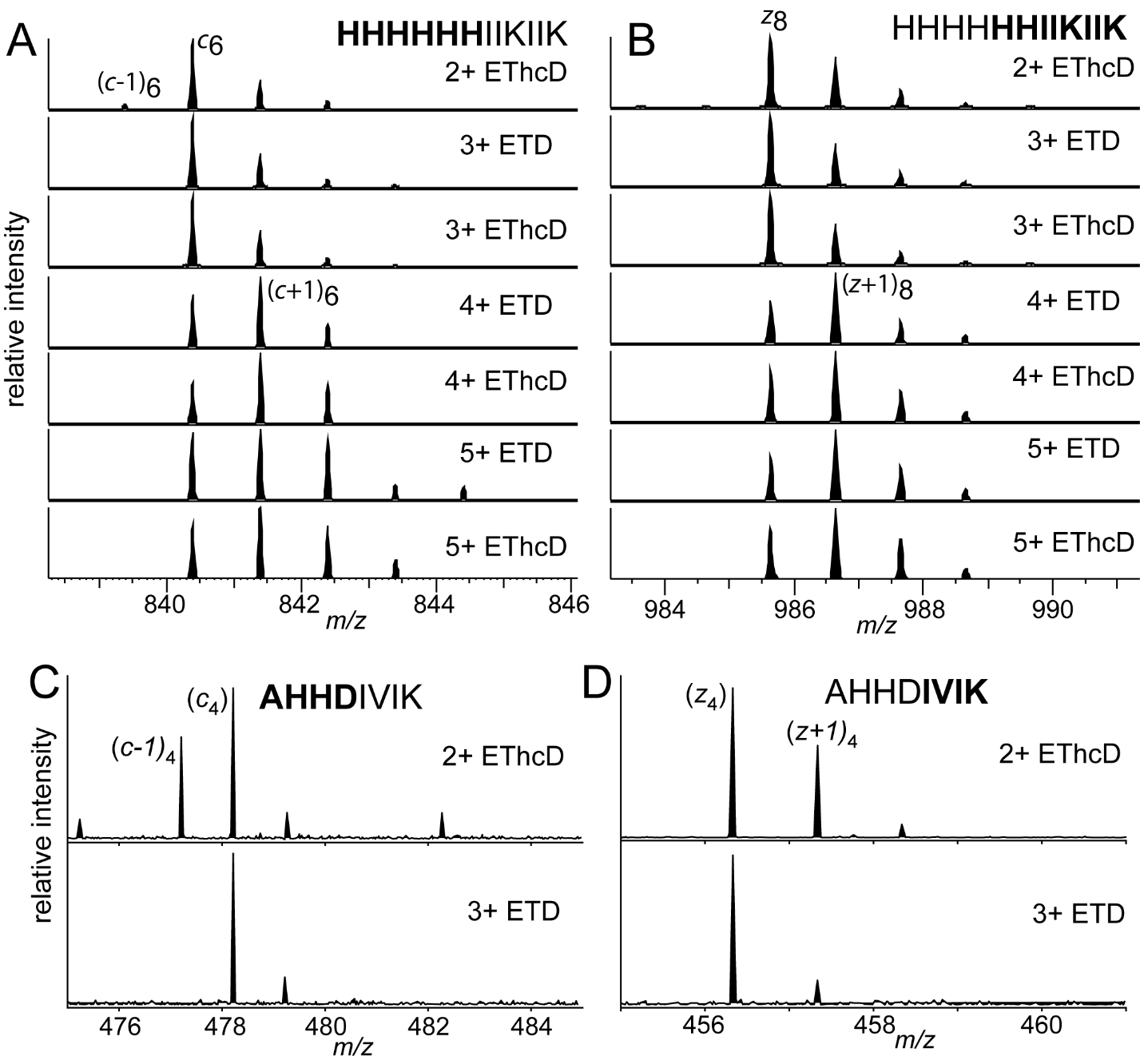


**Figure S2:** Hydrogen transfer products with ETD spectra. **A,B)** Example of the c7 and z8 ion from peptide P1 with either ETD or EThcD from different precursor charge states. C,D) Example of hydrogen transfer products with the *c*_4_ and z_4_ ions from AHHDIVIK from either the 2+ or 3+ precursor ion.

**
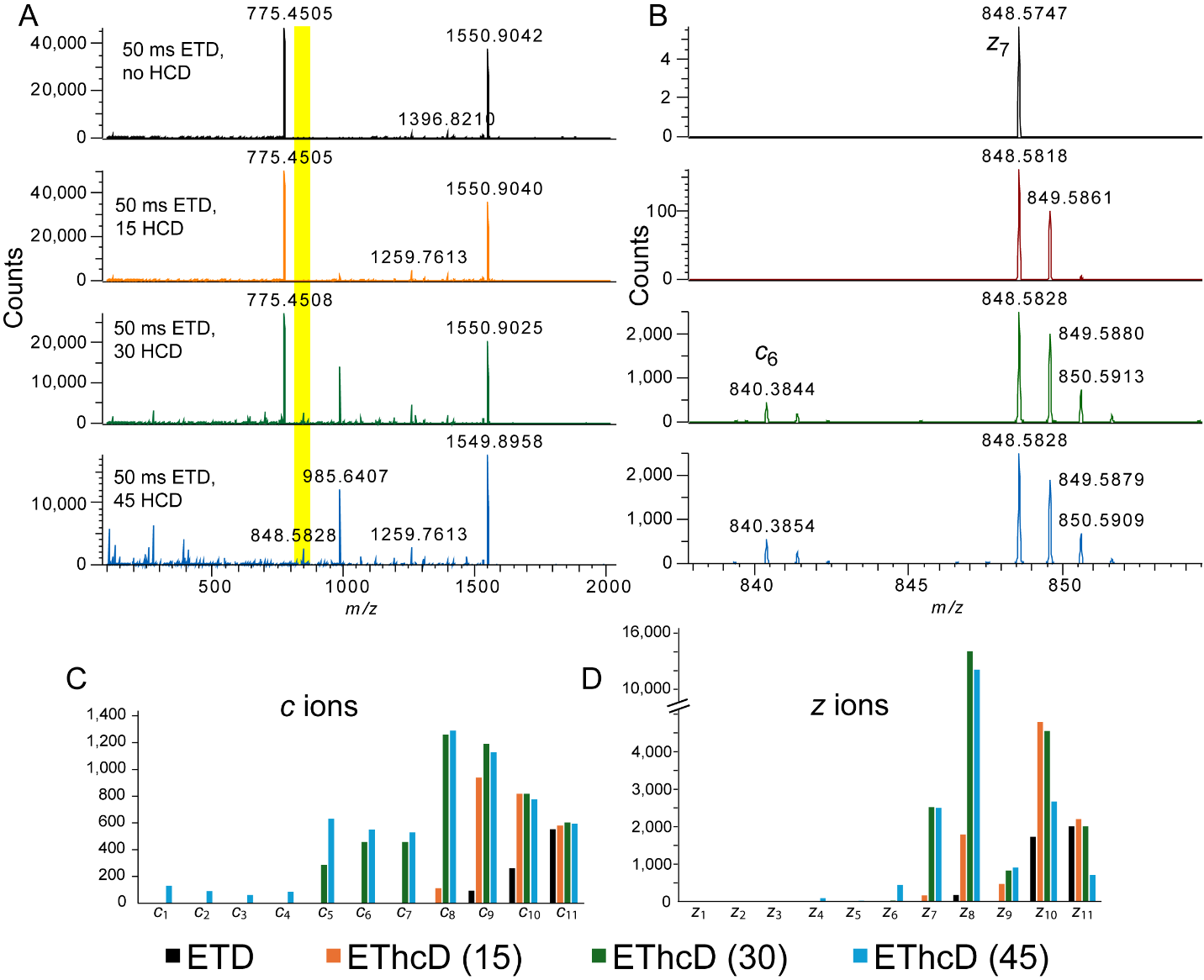
**

**Figure S3:** ETD vs. EThcD fragmentation of the 2+ charge state of peptide P1. A) MS/MS spectra are shown for the 2+ precursor with ETD (no supplemental HCD), or EThcD using 15, 30, or 45% normalized supplemental HCD. B) Zoom in of the m/z region containing the *c*_6_ and *z*_7_ ions. The intensities for all c and z ions with different ETD/EThcD settings are shown in (C) and (D).

**
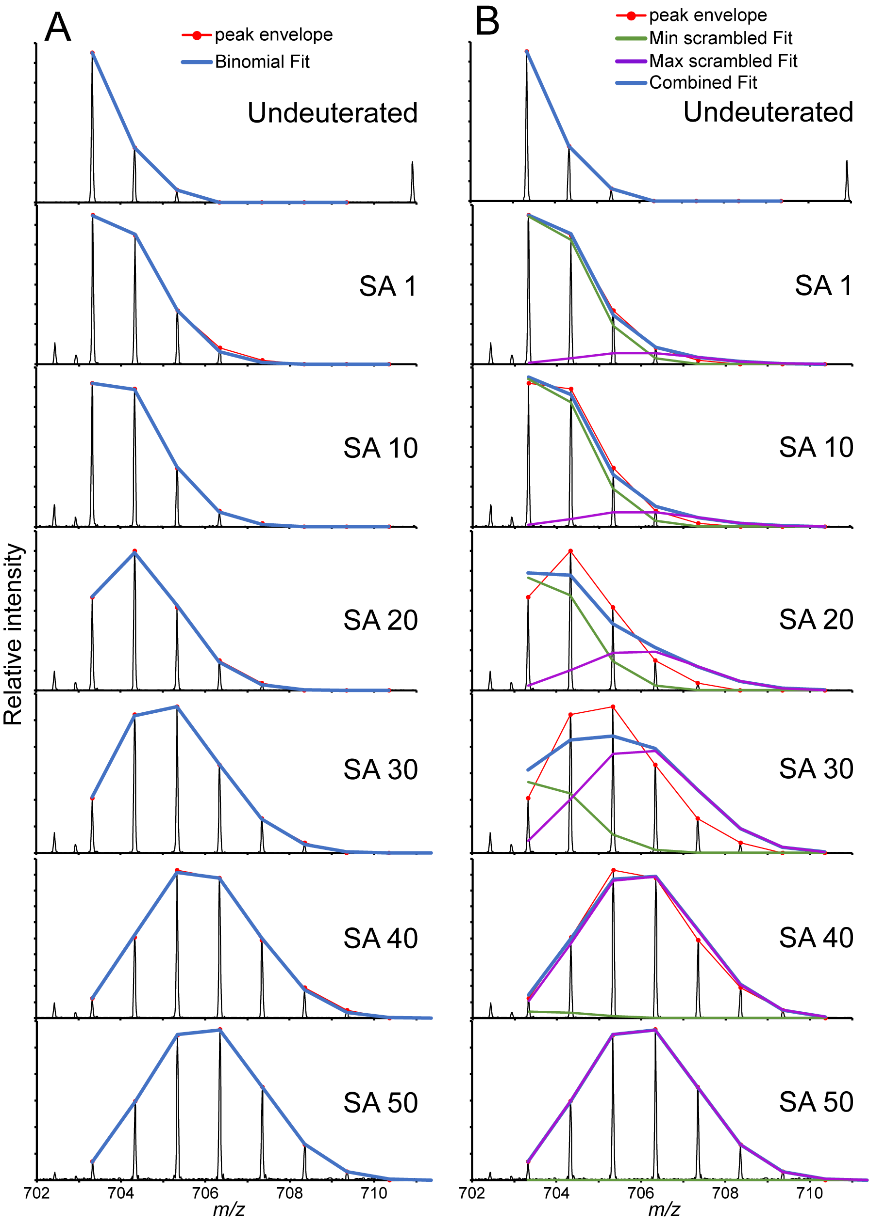
**

**Figure S4:** Isotopic envelopes are shown for the *c*_5_ ion from the ETD spectra of the 3+ charge state of peptide P1 either as the undeuterated reference (top) or with C-terminal amide deuteration at various source activation energies (SA). A) The isotopic envelopes were fit to a single binomial distribution in HX-Express v3, and in all cases a single distribution could encompass the full isotopic envelope. B) A two-state fitting model was attempted, where a linear combination of the isotopic envelopes for the minimum scrambled (green) and maximum scrambled (purple) distributions were applied. At intermediate scrambling levels (SA 20-30), even the best potential fits are inconsistent with the observed data.

**
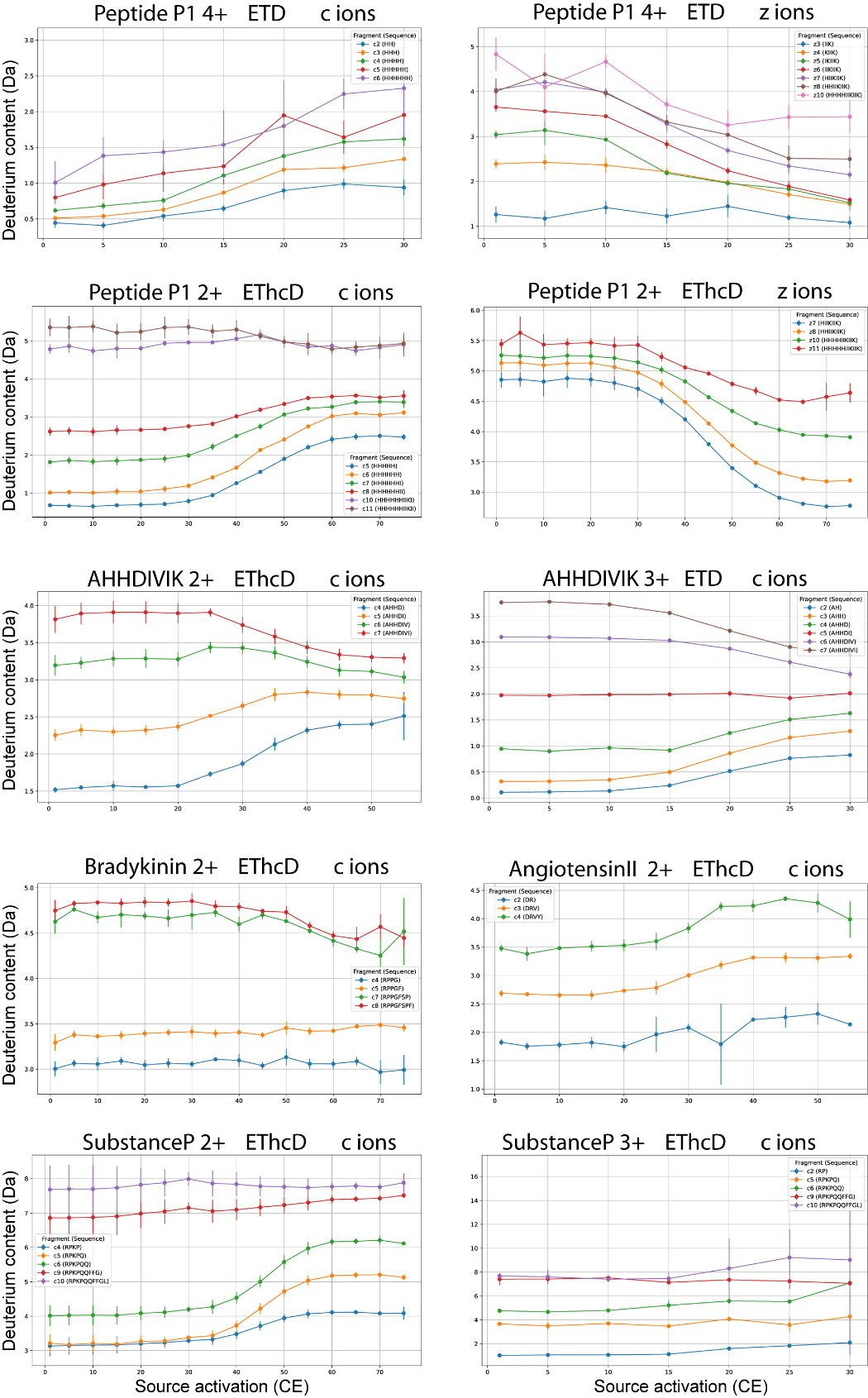
**

**Figure S5:** Scram_50_ analysis for the panel of peptides in this study. Ions monitored are indicated within the insets. Error bars show standard deviations from triplicate measurements.

**
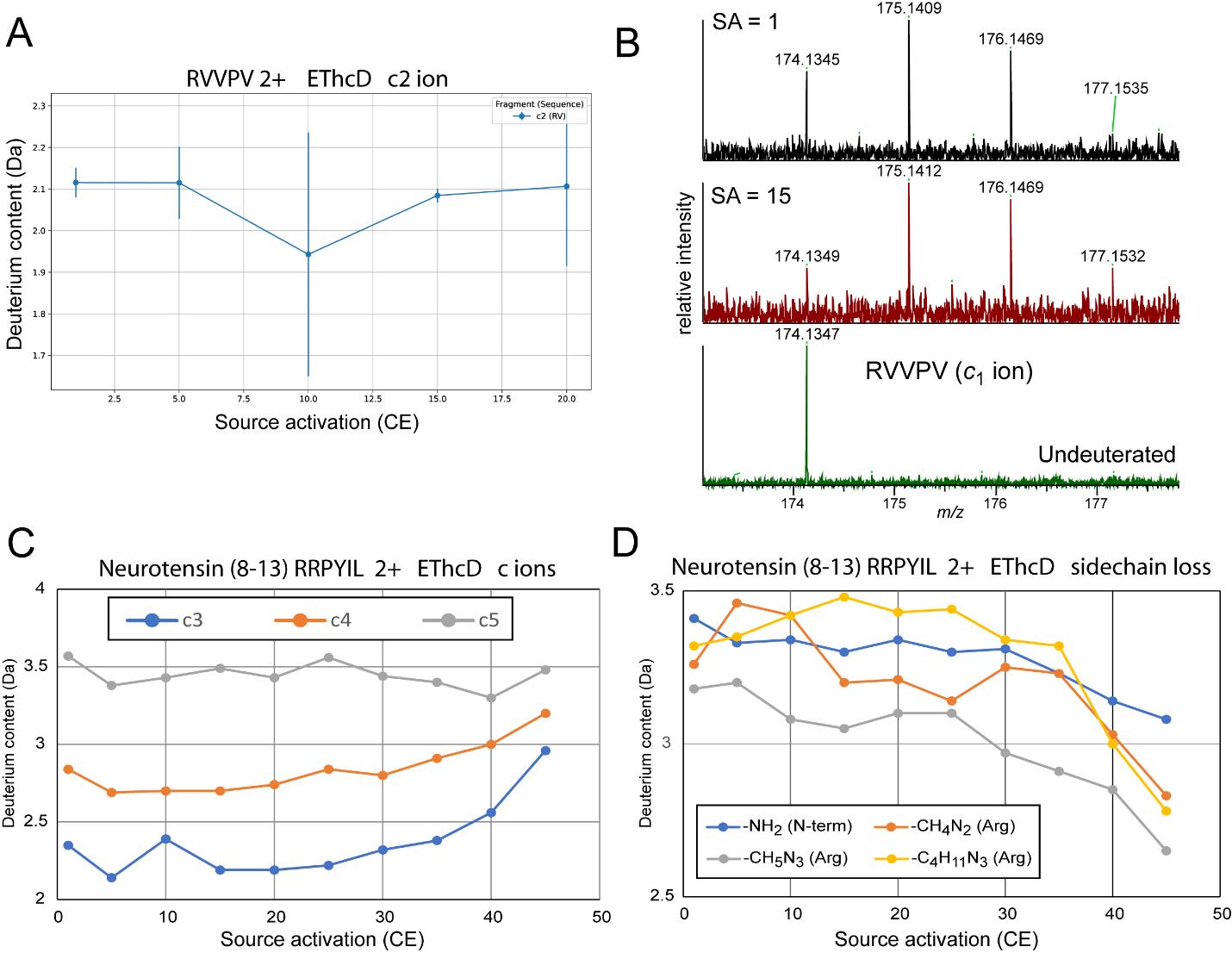
**

**Figure S6:** EThcD analysis of peptide RVVPV and neurotensin 8-13. **A)** The *c*_2_ ion was monitored for deuterium scrambling through a range of source activation energy up to 20, after which the signal was lost as the peptide underwent fragmentation. **B)** Spectra showing the *c*_1_ ion with either no source activation (SA=1) or the highest level before the signal was lost (SA=15). **C, D)** EThcD analysis of neurotensin showing either the deuterium uptake of c ions **(C)** or sidechain losses **(D)** as a function of source activation energy.

**
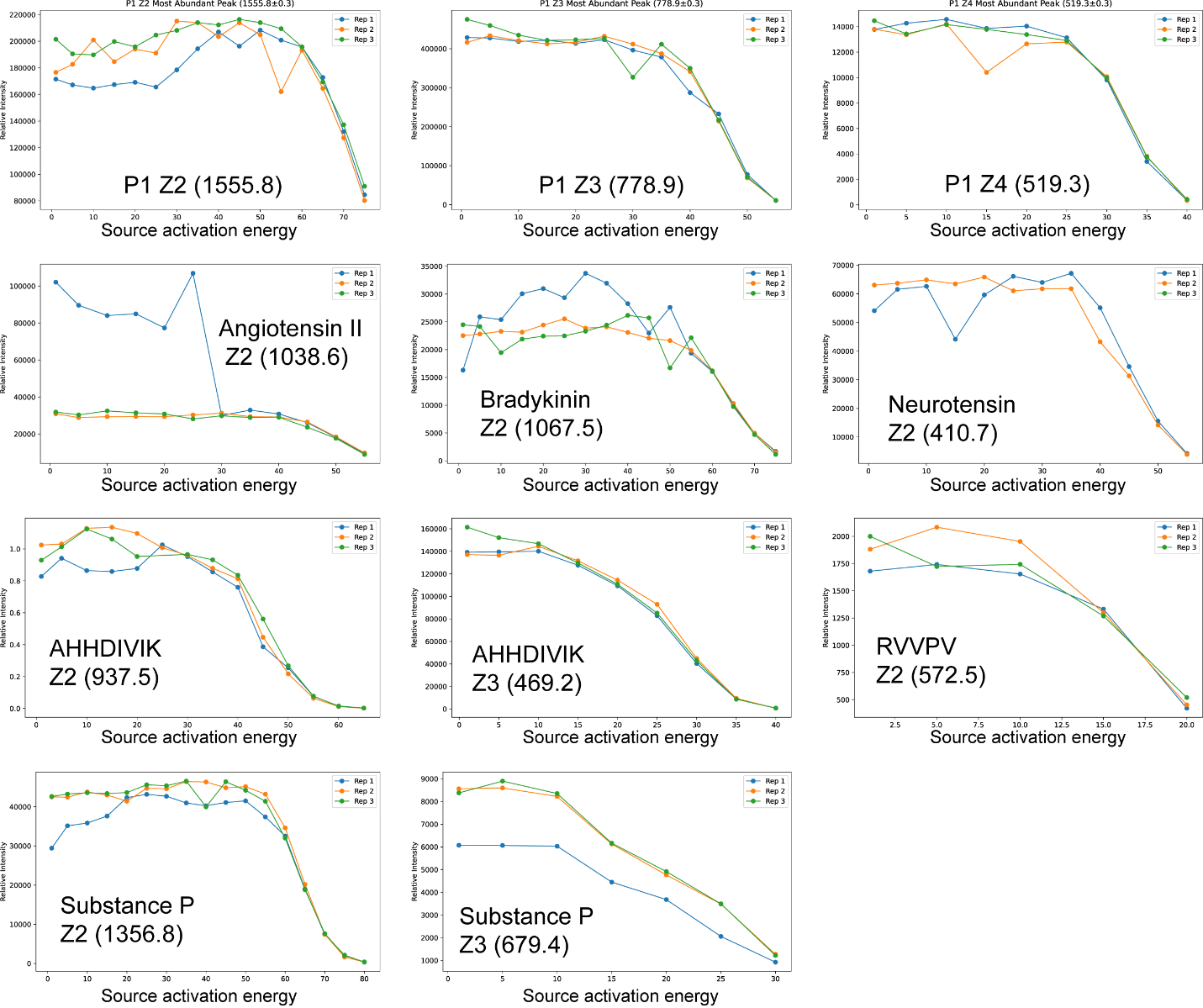
**

**Figure S7:** Mapping of fragmentation thresholds for the panel of peptides. The intensity of the selected precursor ion signal (m/z indicated in parentheses) as a function of source fragmentation energy. Replicate experiments are shown in three different colors.

**Table S1**

| **Section** | **Parameter** | **Default*** | **Optimized** |
| --- | --- | --- | --- |
| Edit Source Offset | Normal | 17.004 | 12 |
| Edit FIRM Offset | FHCD_Inject_spm | -5.692 | -2.4 |
|  | FHCD_To_CT_spm | 4.342 | 2.34 |
| Edit HPT Center Section | LT_Inject | -12 | -12 |
| Edit IRM Offset | CT_To_HCD_spm | -5.541 | -2.54 |
| System - Orbitrap - Utilities | Toggle Full profile data | Off | On** |

Optimized ion transmission settings for minimal scrambling on the Thermo Orbitrap Ascend in peptide mode. The upcoming release of Thermo ICSW 4.3 will be a “Fragile Ion Optics Calibration” function to automatically tune settings for gentle ion transmission and minimal scrambling.

*Default values will vary slightly between instruments and may change with calibrations.

**Full profile data was only used for weaker spectra as it generates data files that are much larger.
